# A new ‘acanthothoracid’ placoderm from the Arctic Canada (Early Devonian) and its bearing on the evolution of jaws and teeth

**DOI:** 10.1098/rsos.250837

**Published:** 2025-09-03

**Authors:** Sébastien Olive, Ilias Kotoulas, Daniel Goujet, Philip C. J. Donoghue, Federica Marone, Martin Rücklin

**Affiliations:** ^1^ Department of Understanding Evolution, Naturalis Biodiversity Center, Leiden, The Netherlands; ^2^ OD Earth and Life History, Royal Belgian Institute of Natural Sciences, Brussels, Belgium; ^3^ Department of Freshwater and Oceanic Science Unit of Research, Laboratory of Ecology and Conservation of Amphibians, Liège University, Liège, Belgium; ^4^ Department of Biology, University of Antwerp, Antwerp, Flanders, Belgium; ^5^ Muséum National d'Histoire Naturelle, Paris, Île-de-France, France; ^6^ Bristol Palaeobiology Group, School of Earth Sciences, University of Bristol, Bristol BS8 1TQ, UK; ^7^ Department of Swiss Light Source, Paul Scherrer Institut, Villigen, Switzerland; ^8^ Institute of Biology, University of Leiden, Leiden, Zuid-Holland, The Netherlands

**Keywords:** 'acanthothoracid', placoderm, Devonian, jaws, *Romundina*, teeth

## Abstract

The origin of jaws and teeth represents one of the most formative episodes in our own evolutionary history. However, this event is poorly understood because of a lack of detailed knowledge of key lineages, including the ‘acanthothoracid’ placoderms, which were among the earliest jawed vertebrates. Here, we describe *Romundina gagnieri* sp. nov., a new species of ‘acanthothoracid’ from the Early Devonian of Arctic Canada. The new species displays anterior supragnathal plates with teeth that we have characterized using synchrotron tomography. Our study shows that teeth are arranged in a concentric manner and that the pattern of tooth addition is centrifugal, including an anterior addition. Overgrowing odontodes, present on the anterior part of the gnathal plates, are covering teeth that can display an hypermineralized layer (probably reflecting the earliest stage of teeth during the ontogeny) or be partially broken. These overgrowing odontodes develop in successive steps and without obvious organization. The presence of a pair of anterior supragnathal plates on the ethmoid part of the endocranium, as well as the growth process of these plates in *R. gagnieri* sp. nov. are similar to the conditions seen notably in arthrodires, compatible with an ancestral gnathostome ancestral condition.

## 1. Introduction

Jaws armed with teeth have long been considered key innovations that underpinned the evolutionary and ecological diversification of jawed vertebrates, though the timing and sequence of origin of these evolutionary novelties has been the subject of considerable debate [1–17]. Although it has been argued that teeth evolved before jaws to which they were recruited convergently in derived placoderms, acanthodians, chondrichthyans and osteichthyans [1], it appears that placoderms, the phylogenetically earliestdiverging lineage of jawed vertebrates, already possessed jaw-bearing teeth [1,2,14,17]. Thus, attempts to elucidate the evolutionary origin of teeth and the nature of their development is to be sought in this paraphyletic [12,18–20] or monophyletic [21,22] assemblage of extinct jawed vertebrates. Although the phylogenetic coherence of Placodermi is debated, it is commonly accepted that the antiarch and ‘acanthothoracid’ placoderm lineages are among the earliest branching lineages of placoderms and jawed vertebrates [12,22]. However, these views are poorly justified, reflecting the absence of a robust phylogeny for early jawed vertebrates. Antiarchs may lack a dentition because of loss [2], but ‘acanthothoracids’ are known to possess dentitions and so, through comparison to other gnathostomes, they may offer unique insights into the nature of the primitive vertebrate dentition.

‘Acanthothoracids’ are scarce in the fossil record [5,12,23–36]. To date, ‘acanthothoracid’ dentitions are known from one unnamed specimen from Arctic Canada that possesses an anterior supragnathal plate [1,5,6,10,12,37] and from three taxa from Czechia [12]. These data have been interpreted to indicate that ‘acanthothoracid’ dentitions are most similar to chondrichthyans and osteichthyans in that the teeth are added posteriorly (lingually) and the dentition is marginal, carried by a cheekbone or a series of short dermal bones along the jaw edges [12]. As such, these characteristics have been interpreted as primitive for all jawed vertebrates [12] though Brazeau *et al*. [16] contend that this view is based on a misinterpretation of skeletal elements of a *Radotina* specimen on which it is based. Here we contribute to the debate with the description of the hitherto unnamed acanthothoracid taxon from Arctic Canada, described and named based on new and previously published material. The dentition is investigated using synchrotron radiation X-ray tomographic microscopy [38] to contribute new data to debate over the evolution of teeth and jaws within gnathostomes.

## 2. Material and methods

### 2.1. Origin of the fossil material

**Figure 1.**
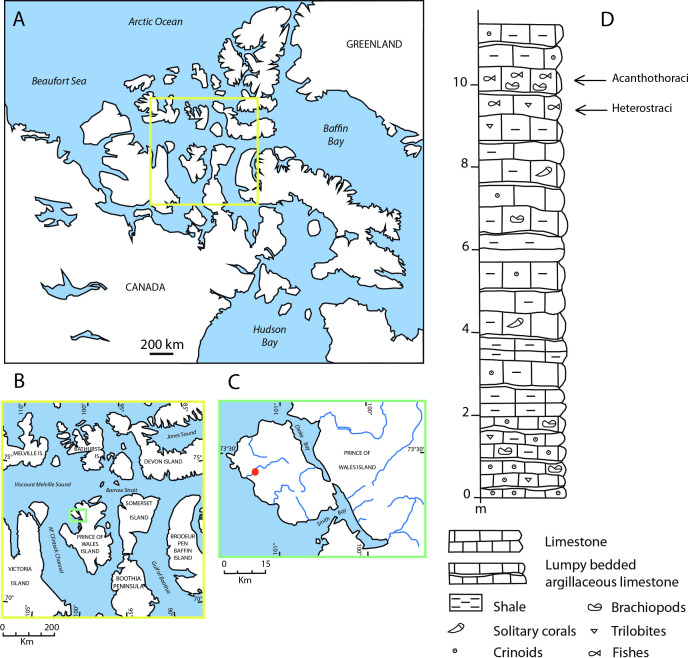
Geographical and stratigraphical localization of *R. gagnieri* sp. nov. specimens. (A) Localization of the Arctic Archipelago. (B) Close-up on the Prince of Wales and surrounding islands. (C) Close-up on the area with the fossiliferous locality (red point). (D) Stratigraphical section of the fossiliferous locality with the two calcareous layers having yielded ‘acanthothoracids’ and heterostracans.

MNHN.F.CPW9 represents a prenasal region and MNHN.F.CPW30 an incomplete skull, both of an ‘acanthothoracid’ placoderm. The two specimens were collected from the Early Devonian (Lower Lochkovian) Drake Bay Formation of the Prince of Wales Island, Arctic Canada (figure 1A,B). The locality marks an isolated stream outcrop approximately 12 m thick [39] (figure 1) and fish remains were found in two calcareous layers at the top of the sequence (figure 1D). ‘Acanthothoracid’ specimens, including the new taxon and specimens of *R. stellina*, were found mainly in the uppermost calcareous layer, whereas the calcareous layer immediately below yielded mainly heterostracans. MNHN.F.CPW9 and MNHN.F.CPW30 were found in 1995 by a Franco-Canadian team (P. Y. Gagnier, D. Goujet, Z. Johanson, A. Lindoe and D. Meckert). All necessary permits were obtained for the described study, which complied with all relevant regulations (mission to Prince of Wales Island funded by UNESCO IGCP 328, MNHN Paris and Institute of Northern Studies: Polar Continental Shelf Project no. 606-95). Specimens are deposited in the Collections of Paleontology of the Muséum National d’Histoire Naturelle, Paris (MNHN), France.

### 2.2. Chemical and virtual preparations

The two specimens were originally enclosed in a limestone matrix. Preparation entailed mounting the specimens on resin blocks, followed by dissolution of the external limestone matrix with 8% formic acid buffered with tricalcium diphosphate; most of the matrix inside the skull remains intact. This has preserved the delicate internal perichondral ossifications, which would otherwise have collapsed.

**Figure 2.**
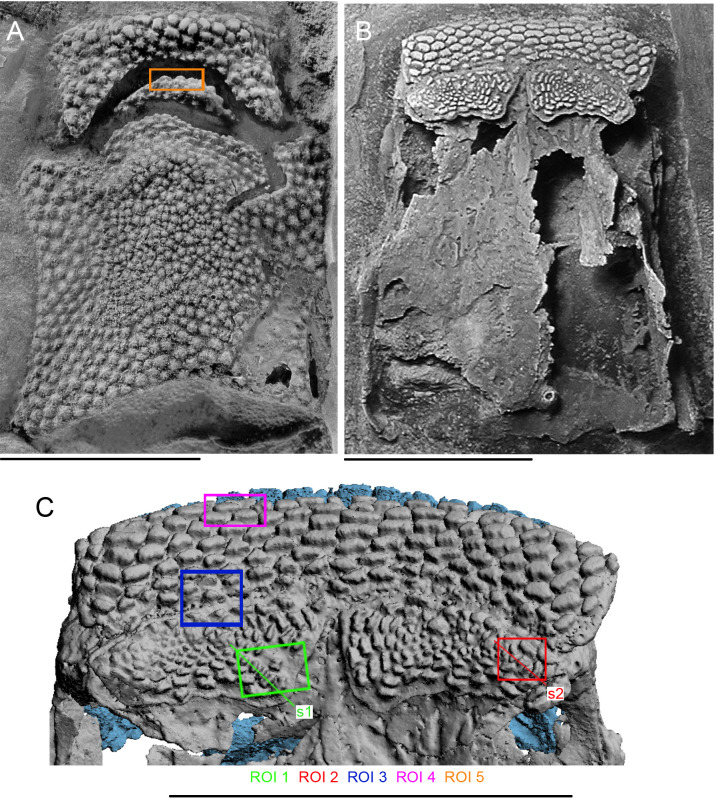
*R. gagnieri* sp. nov., prenasal area, MNHN.F.CPW9. (A) MNHN.F.CPW9a, dorsal view, photograph. (B) MNHN.F.CPW9b, ventral view with the gnathal plates, photograph. (C) Volume rendering of microtomography data of the anteroventral part (models of CPW9a and b have been fused) with localization of the different regions of interest (ROI, ‘s’ for slice). Blue for CPW9a, grey for CPW9b. Scale bars equal 1 cm.

MNHN.F.CPW9 was reconstructed using computed tomography (figure 2) based on raw X-ray projection data from Smith *et al*. [37] available in the Dryad Data Repository [40]. Smith *et al.* [37] scanned the specimen using a Nikon metrology HMX ST 225, with a 1.5-mm copper filter at 200 kV, at the Imaging and Analysis Centre, Natural History Museum, London. MNHN.F.CPW9a and b were scanned independently. Voxel dimensions for CPW9a are 0.017 mm and the number of slices is 1478. Voxel dimensions for CPW9b are 0.020 mm and the number of slices is 1414. These data were segmented using MIMICS (Materialise Interactive Medical Image Control System) 21.0 (Materialise NV). Part and counterpart were merged using 3-matic Research 13.0 (Materialise NV).

Five regions of interest (ROI) on MNHN.F.CPW9 (figure 2) were characterized using synchrotron radiation X-ray tomographic microscopy (SRXTM) at the TOMCAT (X02DA) beamline of the Swiss Lights Source, Paul Scherrer Institute, Switzerland [41]. These regions of interest were scanned with 1501 (ROIs 2 and 4) or 1001 (ROIs 1, 3 and 5) projections distributed equi-angularly over 180°, using a 10× objective, resulting in a dataset with voxel dimensions of 0.65 µm. The measurement energy was 35 (ROIs 1, 3 and 5) or 32 (ROIs 2 and 4) keV with an exposure time of 1800 (ROIs 1, 3 and 5) or 2000 (ROIs 2 and 3) ms. Phase retrieval was used applying a Paganin algorithm [42] to the corrected projections and consequently tomograms were reconstructed for all ROIs. The raw slice data are available in the Dryad Data Repository [40]. These data were analysed using AVIZO 2019.3 (https://www.fei.com). Volume rendering of odontodes was made, slice by slice, from the tip to the base.

MNHN.F.CPW30 was scanned using a Zeiss Xradia 520 Versa micro-CT scanner, 50 kV (Naturalis Biodiversity Centre, Leiden). MNHN.F.CPW30 is preserved in two parts. CPW30a corresponds to an almost complete endocranium, whereas CPW30b represents only a fragment of it (of the left part). MNHN.F.CPW30a and b were scanned independently, with the voxel dimensions of 0.023 and 0.012 mm for CPW30a (number of slices of 1018) and CPW30b (number of slices of 1015), respectively. Both datasets were merged virtually and segmented using AVIZO 2019.3. The raw slice data are available in the Dryad Data Repository [40]. The endocranial cavities and the cavities of the nerve canals were segmented, but it should be noted that this does not represent the actual shape and size of the brain and nerves, rather the dimensions of their cavities. In extant species, the volume of the braincase that the brain occupies can be significantly less than the volume of the endocranial cavity, reaching as little as 1% in the case of the coelacanth *Latimeria* [43].

### 2.3. Terminology

We follow the terminology adopted by Huysseune *et al*. ([44], tab. 1) based on the works and definitions given by Ørvig [45,46], Reif [47], Donoghue [48] and Donoghue & Rücklin [11] for characterizing odontodes and related tissues. Thus, teeth correspond to internal odontodes and odontodes located in the skin to external/dermal odontodes. We refer to odontodes that overgrow teeth and colonize the oral cavity as overgrowing odontodes in the text.

### 2.4. Anatomical abbreviations

III, oculomotor nerve; IV, trochlearis nerve; V1, profundus ramus of trigeminal nerve; V2, maxillary ramus of the trigeminal nerve; V3.s.p, ramus of the trigeminal nerve connected to the sensory pits; V3.soc, ramus of the trigeminal nerve connected to the supraorbital sensory line; VI, abducens nerve; VII.hm, hyomandibular ramus of the facial nerve; VII.lc, ramus of the facial nerve connected to the lateral sensory line; VII.pal, palatal ramus of the facial nerve; VIII, acoustic nerve; X, vagus nerve; X.lc, ramus of the vagus nerve connected to the lateral sensory line; X.ppl, ramus of the vagus nerve connected with the posterior pitline; spi1−2, spinooccipital nerves; ?, unidentified foramina in the ethmoid region; amp.a, anterior ampulla of the otic capsule; amp.e, external ampulla of the otic capsule; aut.art, articulation area for the autopalatine, bu, perichondral bulge supporting the anterior supragnathal plates; csa, anterior semicircular canal, csc, central sensory line groove; cse, external semicircular canal; csp, posterior semicircular canal; d.end, endolymphatic duct foramina; d.myo, dorsal myodome; d.myo.IV, dorsal myodome for the trochlearis-innervated eye muscle; end.fl, floor of the endocranium; es, eyestalk; eth.lc, ethmoid lateral line commissure; eth.lc.ae; anterior expansion of the ethmoid lateral commissure; f.III, foramen for the oculomotor nerve; f.j.v, foramen for the jugular vein; f.IV, foramen for the trochlearis nerve; f.V1, foramen for the first branch of the trigeminal nerve; f.VI, foramen for the abducens nerve; g.pse.a, groove of the pseudobranchial artery; hyp, hypophysis; hyp.f, hypophysial fenestra; hyp.v, hypophysial vein; ifc, infraorbital sensory line groove; j.v, jugular vein; lc, main lateral sensory line groove; n, notch on the perichondral bone; ot.v?, possible otic vein; p.myo, posterior myodome; pit.v, pituitary vein; pmc, postmarginal sensory line groove; ppl, posterior pitline; Prm, premedian plate; rec.V, trigeminal recess; soc, supraorbital sensory line groove.

## 3. Results

### 3.1. Systematic palaeontology

Class PLACODERMI McCoy, 1848
Order ACANTHOTHORACI Stensiö, 1944
Family PALAEACANTHASPIDAE Stensiö, 1944
Genus *Romundina* Ørvig, 1975

*Emended diagnosis*. ‘Acanthothoracid’ placoderm with a subdivision of the endocranium into separate premedian-ethmoid and orbito-otic-occipital areas. Presence of a large, posterior myodome in each orbital cavity. On the lateral face of the premedian-ethmoid region, presence of large areas of articulation with the autopalatine part of the palatoquadrate. Ornamentation with stellate semidentine odontodes.

*Type Species*. *R. stellina* Ørvig, 1975

*R. gagnieri* sp. nov.
*Romundina* sp. [17]
Acanthothoracid placoderm [5]
*Romundina* sp. [6]
*Romundina* [10]
Acanthothoraci [37]
*Romundina* [49]
Acanthothoracid placoderm [12]

*Holotype.* MNHN.F.CPW9a and b (part and counter-part): prenasal region.

**Figure 3.**
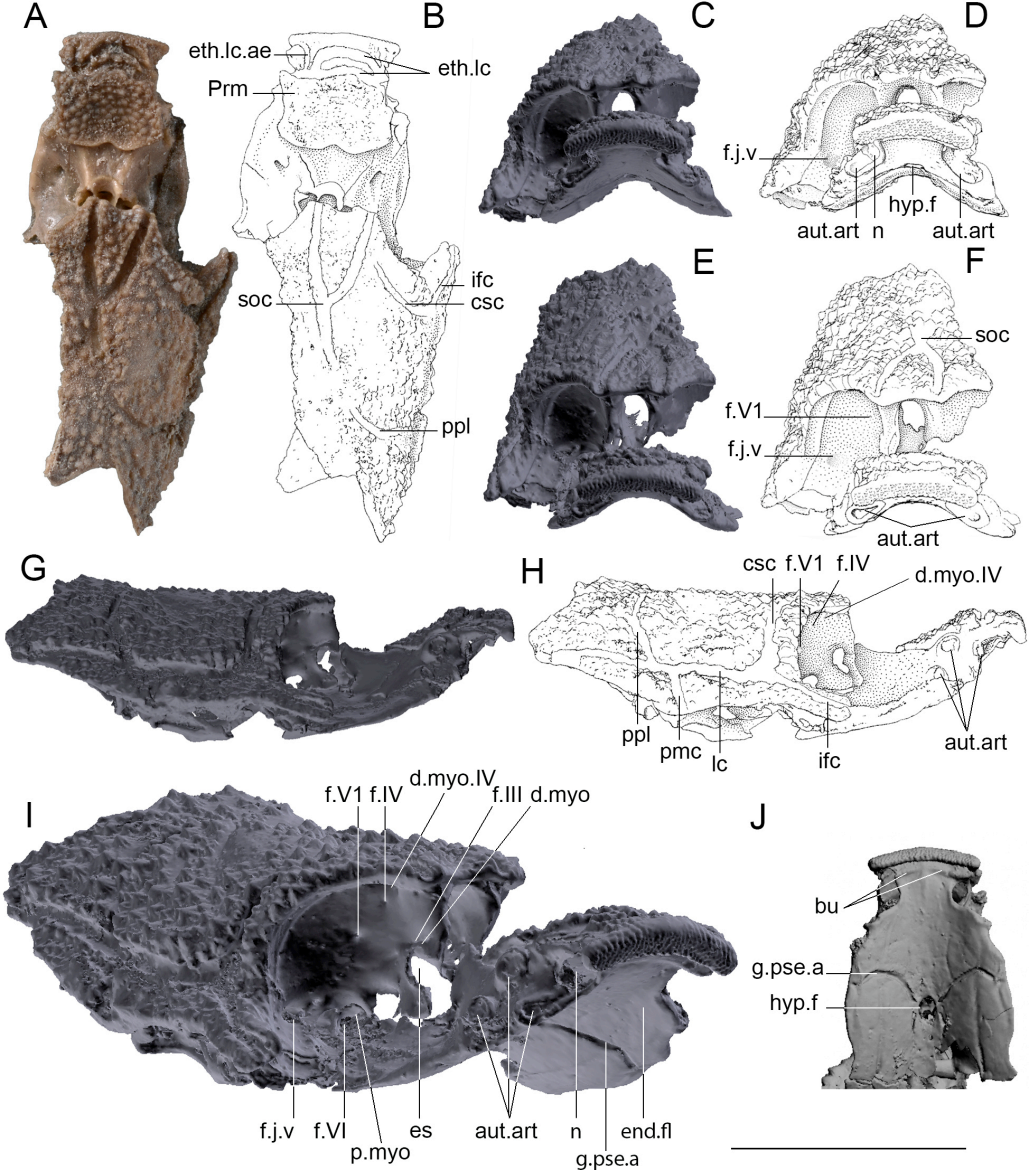
*R. gagnieri* sp. nov., incomplete skull, MNHN.F.CPW30. (A) Dorsal view, photograph. (B) Dorsal view, interpretative drawing. (C) Anterior view, volume rendering of microtomography data. (D) Anterior view, interpretative drawing. (E). Dorso-anterior view, volume rendering of microtomography data. (F) Dorso-anterior view, interpretative drawing. (G). Right lateral view, volume rendering of microtomography data. (H) Right lateral view, interpretative drawing. (I) Right dorso-anterior lateral view, volume rendering of microtomography data. (J) Ventral view of the orbital region, volume rendering of microtomography data. Scale bar equals 1 cm for A–H, J and 0.6 cm for I.

*Other material.* MNHN.F.CPW30a and b (part and counter-part): incomplete skull showing the premedian-ethmoid area and parts of the orbital, otic and occipital areas. This specimen is slightly distorted in the area between the premedian plate and the orbital area, giving the impression that the specimen is asymmetric (e.g. figure 3A).

*Etymology.* In honour of Dr Pierre-Yves Gagnier, who greatly improved our knowledge of early vertebrates and who was part of the expedition to Prince of Wales Island in 1995.

*Type locality.* Locality 10 [39] in Prince of Wales Island, Canadian Arctic Archipelago.

*Type horizon*. Drake Bay Formation. Early Lochkovian.

*Diagnosis.* Skull laterally compressed, and skull floor strongly vaulted. Elongated quadrangular premedian plate with an anterior expansion extending ventrally. Orbital area constricted laterally. Orbital and premedian-ethmoid areas dorsally oriented. Ethmoid lateral line commissure, on the premedian plate, divided in two branches and located in the first anterior third of the plate. Supraorbital canals joining posteriorly and forming a single canal. Central sensory line groove parallel to the dorsal posterior margin of the orbit and parallel to the posterior pitline.

*Remarks*. MNHN.F.CPW30 and 9 are assigned to the same species because of the elongated and quadrangular shape of the premedian plate, the ethmoid lateral line commissure that is splitting into two, and the similar ornamentation.

### 3.2. Description

#### 3.2.1. Skull roof

**Figure 4.**
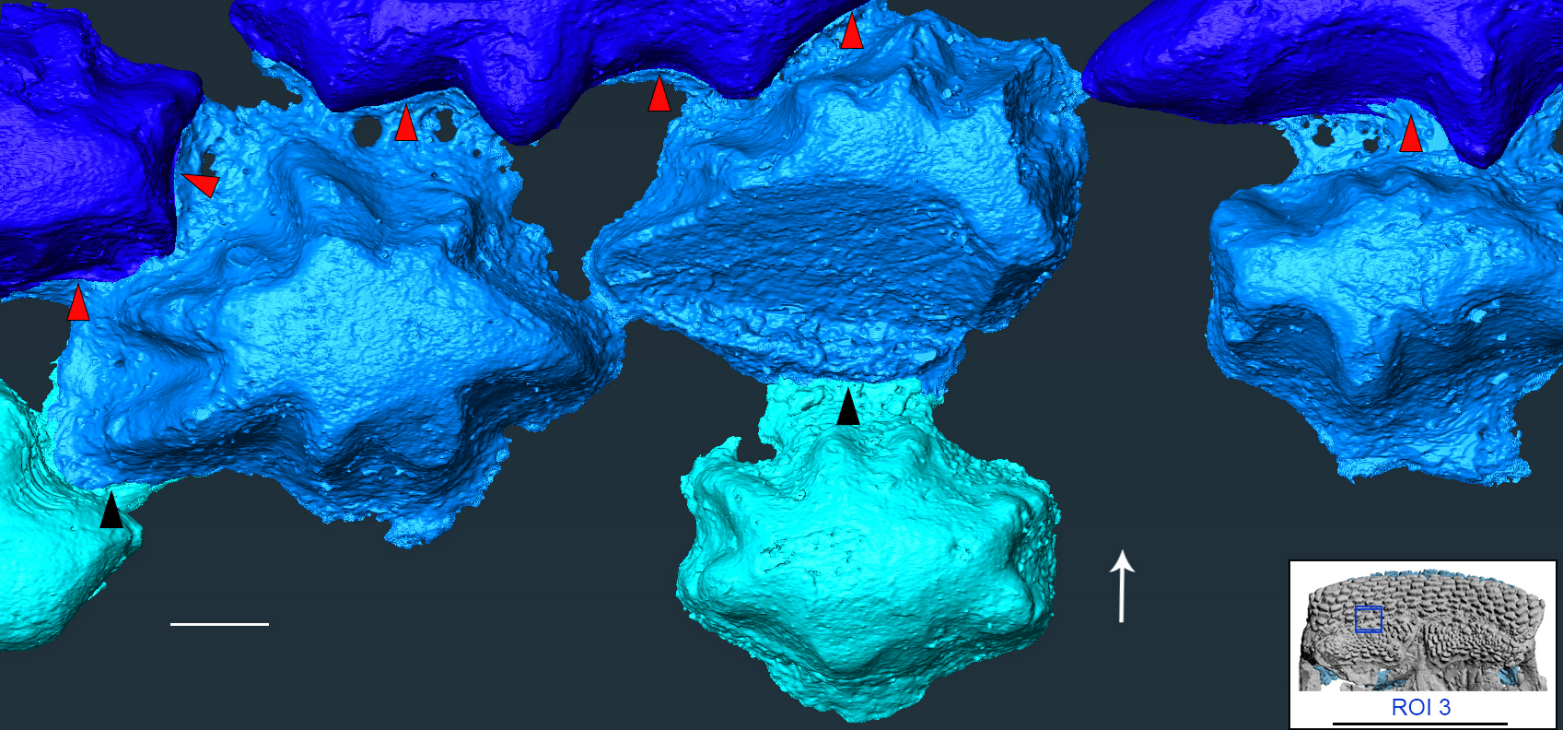
*R. gagnieri* sp. nov., volume rendering of SRXTM data of the posterior portion of the ventral expansion of the premedian plate (anterior area of ROI 3). Black represents parts of the premedian plate that were not volume rendered in detail. The three types of blue correspond to three generations of odontodes (dark blue being the oldest and cyan the youngest). Red arrow tips point the overlap areas of light blue odontodes on dark blue ones. Black arrow tips point the overlap areas of cyan odontodes on light blue ones. White arrow points anteriorly. Scale bar equals 0.1 mm. Miniature, on the bottom right, from figure 2 to locate ROI 3 (scale bar equals 1 cm).

The *premedian plate* (Prm, figure 3) is known from a juvenile (MNHN.F.CPW30) and an adult (MNHN.F.CPW9) specimen. It is elongated, quadrangular and anteroventrally expanded; slightly in the juvenile and more ventrally developed in the adult specimen. The frontal torus is rounder in the juvenile skull (figure 3J), whereas it is slightly flattened in the larger specimen ([12]: fig. S2D). The external surface is concave on the juvenile specimen and convex in the adult one. The posterior margin of the premedian plate is (i) strongly concave in the adult specimen and (ii) with a small medial embayment in the juvenile specimen. This area was in contact with the rostronasal capsule and the anterior process of the capsule might be in contact with the embayment in the juvenile and the larger concave area in the adult. In both specimens, the ethmoid lateral line commissure (eth. lc, figure 3) is divided in two branches, parallel to the anterior margin, and joining laterally. On MNHN.F.CPW9, there is an extra branch oriented obliquely posteriorly. On MNHN.F.CPW30, there is a short anterior expansion of the lateral line commissure on the left side (eth. lc. ae, figure 3). The premedian plate is divided in two parts: (i) the anterior part, which bears the ethmoid lateral line commissure, and displays concave lateral margins, and (ii) the posterior part with concave lateral margins too. The posterior part of the premedian plate represents two thirds of the total length of the adult premedian plate (MNHN.F.CPW9; figure 2A,B), whereas it accounts for slightly more than half of the juvenile premedian plate (MNHN.F.CPW30; figure 3A,B). This suggests that the posterior part of the plate mainly elongates during growth. On the smaller specimen, external odontodes on the anteroventral margin are smaller than the ones on the dorsal part of the premedian plate. On the bigger specimen, external odontodes are smaller in the dorso-central part of the premedian plate and more numerous and larger in anterior, lateral and posterior areas, indicating an early growth in the central area and a later increased growth posteriorly. The growth of external odontodes on the ventral expansion of the premedian plate shows that posterior external odontodes are younger than the anterior ones since the anterior margins of the posterior odontodes overlap the posterior margins of the anteriormost external odontodes (figure 4). Therefore, external odontodes on the premedian plate are added in an oral direction.

The description of the remaining skull roof plates is only possible for the juvenile stage (figure 3). Postorbital plates are present with postorbital processes. The boundaries between the skull roof plates are not discernible, but the sensory line groove pattern is well observable. The supraorbital sensory line grooves (soc, figure 3B,F) join posteriorly on the level of the posterior orbital margin and form a sole canal. The main lateral sensory line groove (lc, figure 3H) runs laterally to the skull roof. Anteriorly, it is divided into two branches: (i) the infraorbital sensory line groove (ifc, figure 3H) running along the ventral margin of the preorbital plate and (ii) the central sensory line groove (csc, figure 3H) running in parallel to the lateral and dorsal margins of the orbit. Posteriorly, there is a short lateral postmarginal sensory line groove (pmc, figure 3H) and a longer posterior pit line (ppl, figure 3H), both running from the main lateral sensory line groove. The posterior pit line stretches towards the centre of the skull roof.

#### 3.2.2. Endocranium: external morphology

MNHN.F.CPW30 displays several areas of the endocranium. Dorsally, the premedian-ethmoid region of the specimen is well preserved. In the orbits and the orbital shelves, the perichondral bone is almost intact, preserving foramina housing the nerves innervating the eye. On the ventral side, the cranial floor is preserved only anteriorly of the orbits.

On the lateral side of the snout, the perichondral bone forms an ovoid notch (n, figure 3D,I). This notch may correspond to the articulation point of the anterior process of the inner side of the suborbital plate of the autopalatine [34]. Further posteriorly from the notch three circular crests are visible that correspond to the points for the articulation of the autopalatine part of the palatoquadrate (aut.art, figure 3D,F,H,I). Posterior to the premedian plate and limited by the orbital margins, the rim carrying the rostronasal capsule, connects the premedian-ethmoid region to the anterior margin of the orbital region.

The orbital region extends from the anterior orbital margin, which connects the premedian–ethmoid region, to the anterior postorbital process. The posterior boundary of the orbital wall houses the foramen of the hyomandibular branch of the facial nerve. Anterolaterally the margin of the orbital region is formed by the suborbital shelves which are relatively wide. The surface of the orbits is covered by perichondral bone, and they open anterolaterally. The orbital region includes articulation points for the eye muscles and foramina for the cranial nerves innervating the eye and the blood vessels exiting the orbital wall. On the anterior part of the orbits, the most prominent feature is the eyestalk (es, figure 3I) which is keyhole-shaped and is oriented dorsoventrally. However, the exact morphology is not visible due to fragmentation in that part of the specimen. Right above the eyestalk due to imperfect preservation, only an impression of the foramen of the oculomotor (f.III) nerve is visible, which lies within the dorsal myodome (d.myo, figure 3I). On the surface of the anteromedial part of the orbital wall, two small foramina corresponding to the trochlear (f.IV, figure 3H,I) and the ophthalmic branch of the trigeminal nerve (f.V1, figure 3G–I) are visible. The ventral part of the orbital wall exhibits two large openings. The most medial one forms a large chamber that is connected with the common foramen of the trigeminal and facial nerves and corresponds to the posterior myodome (p.myo, figure 3I). Within that chamber the opening for the abducens nerve (f.VI, figure 3I) is present. The most lateral opening is identified as the foramen of the jugular vein (f.j.v, figure 3D,F,I). On the anterodorsal part of the orbit, right under the skull roof and above the foramen of the trochlear nerve, a large depression is visible. This depression corresponds to the dorsal myodome for the trochlearis-innervated eye muscle (d.myo.IV, figure 3H,I). The ventral surface of the suborbital shelves exhibits two elongated grooves with an anteroposterior direction that most likely housed the palatine branch of the facial nerve. The floor of the orbital area of the neurocranium displays in its center the hypophysial fenestra (hyp.f, figure 3J). Laterally to the later run two strong grooves for the pseudobranchial arteries (g.pse.a, figure 3J).

#### 3.2.3. Endocranium: internal morphology

**Figure 5.**
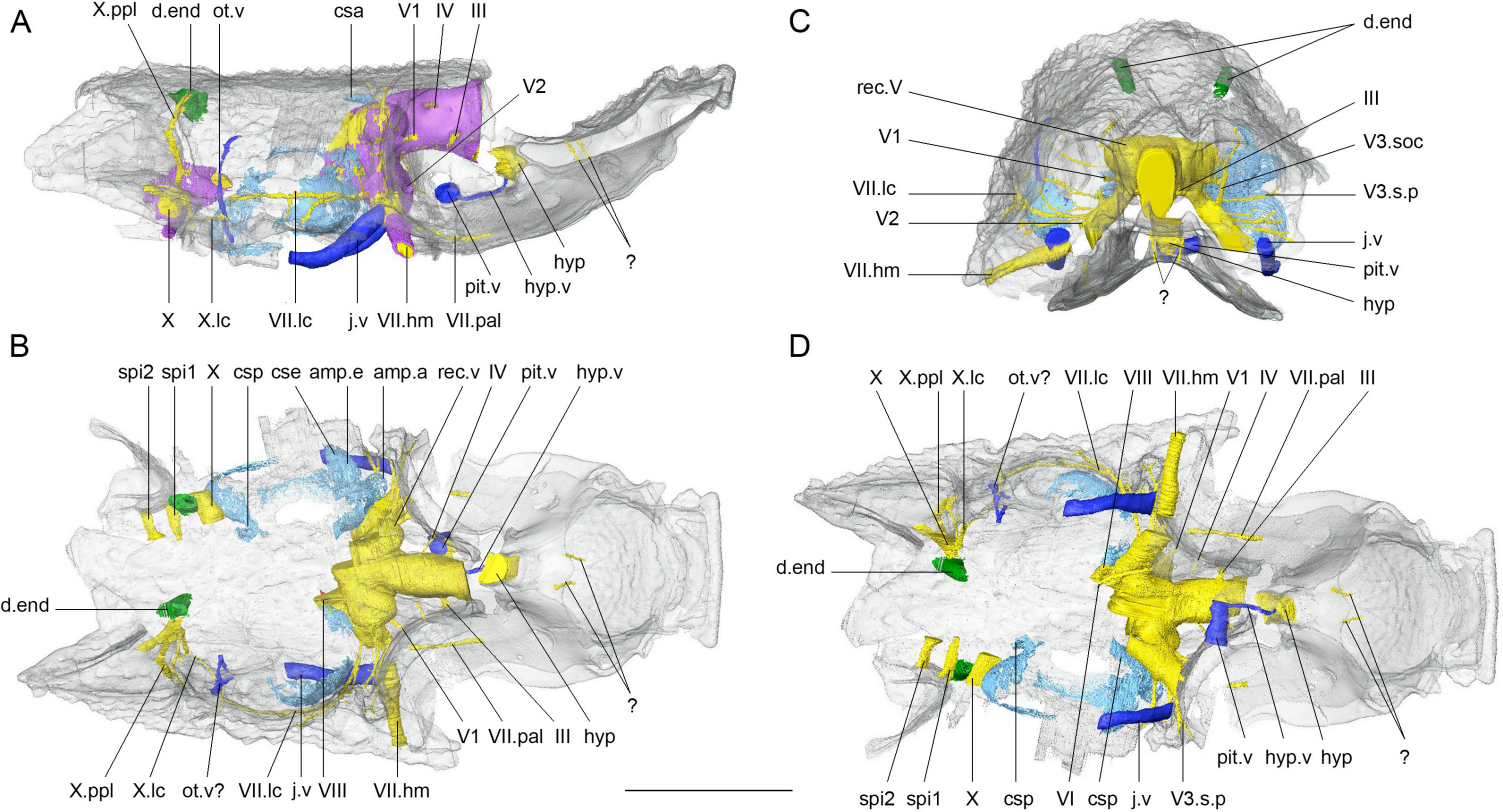
*R. gagnieri* sp. nov., reconstruction of the endocranial cavity, MNHN.F.CPW30. (A) right lateral view. (B) dorsal view. (C) anterior view. (D) ventral view. Scale bar equals 5 mm. Purple for perichondral bone, nervous system in yellow, inner ear organ in light blue, endolymphatic ducts in green and veins in dark blue. Dermal skull roof in semitransparent. Scale bar equals 5 mm.

Anteriorly, in the premedian–ethmoid region, the ethmoid is pierced by a pair of dorsally directed canals (?, figure 5). Its physiological function has not been clarified yet. The preserved endocranial cavity of the specimen starts in the post-ethmoid part of the neurocranium, situated directly between the orbits. On the dorsal and the lateral sides, the perichondral bone covering the endocranial cavity is well preserved, while on the ventral side, it is heavily fragmented. Within the orbital region, the boundary between the diencephalon and the mesencephalon is marked by a slight swelling, anterior to the oculomotor nerve (III, figure 5). The mesencephalic region is shaped as an elliptic cylinder, with lateral sides flatter than the dorsal, which narrows posteriorly to the trochlear nerve (IV, figure 5A,B,D). This narrowing is followed by the bulging of the trigeminal recess, which has a very pronounced spherical shape, grows anterolaterally in two hemispheres and forms a sharp evagination with the lateral walls of the mesencephalon. The part of the brain cavity forming the recess extends up to the anterior otic region. Beyond that point, the posterior part of the endocranial cavity is absent, except for a small lateral fragment on the left side of the anterior occipital region, from which three cranial nerve canals (X, spi1, spi2, figure 5B,D) project laterally.

The canal for the oculomotor nerve (III, figure 5) is situated ventrally on the lateral wall of the brain cavity. Its main body is relatively short, and laterally directed, to the orbital wall, posterior to the eye stalk area. Right before reaching the orbital wall, it divides into two branches. On the surface of the orbital wall, one branch connects to a foramen, most likely belonging to an oculomotorius-innervated eye muscle, homologous to other species of placoderms. The direction of the other branch on the orbital wall is not very well preserved. The trochlear nerve (IV, figure 5A,B,D) exits from the dorsal side of the lateral wall of the brain cavity, anterolaterally directed and connected to a foramen in the dorsal side of the orbital wall.

The trigeminal nerve (V, figure 5) comprises three main divisions, which exit the trigeminal recess from different points, without all three of them joining in the same nerve canal. The profundus branch (V1, figure 5) exits the anterior part of the recess anterolaterally directed and connects to the medial area of the orbital wall. From the left profundus branch, shortly after exiting the recess, an unidentified much thinner canal branches off, dorsally directed, connecting to the dorsal wall of the orbit. The two remaining branches (V2 and V3, figure 5A,C,D) exit posteroventrally from the profundus within a common canal in ventrolateral direction. Right above the posterior myodome, the V2 branch turns anteriorly and connects with the orbital wall. The direction of the V3 branch of the trigeminal nerve is uncertain due to a common fossa created by its connection with the canal of the facial nerve and jugular vein, right behind the posterior myodome. Shortly after the V2–V3 common nerve canal exits the brain cavity, on its dorsal side a thin canal branches off, and bifurcates to a dorsally directed (V3.soc, figure 5C) and a laterally directed (V3.s.p, figure 5C,D) branch. The V3.soc is probably the ramus of the trigeminal nerve connected with the supraorbital sensory line and the V3.s.p is the ramus connected to the sensory pits on the infraorbital sensory line.

The exit point of the facial nerve (VII) from the brain cavity is situated posteroventrally from the trigeminal recess, just anteriorly to the otic region. The facial nerve is ventrolaterally directed and joins the V2 and V3 foramen in a common canal. When it reaches the fossa behind the posterior myodome, it divides into the palatal (VII.pal, figure 5A,B,D) and the hyomandibular (VII.hm, figure 5) branches. The palatal is directed anteriorly, exiting the orbital wall from the ventral side of the posterior myodome, and housed in a groove of the orbital floor bone. The hyomandibular branch runs laterally along the posterior orbital wall and exits from the anterior postorbital process. Close to the exit point of the facial nerve from the endocranial cavity a much thinner branch (VII.lc, figure 5) turns posteriorly, and runs along the lateral sensory line, surrounding the otic capsule, reaching close to the vagus nerve (X, figure 5A,B,D) in the posterior otic region.

The acoustic nerve (VIII, figure 5B,D) exits the endocranial cavity from the posterior side of a small recess, shared with the exit of the facial nerve. The acoustic nerve is very short, laterally directed and connects with the lateroventral part of the otic capsule. Due to the heavy fragmentation of the capsule’s perichondral bone, it is uncertain to which part of the capsule it connects, but most likely to the sacculus. On the ventral side, a thin canal starts very close to the shared recess of the facial and acoustic nerves, most likely for the abducens nerve (VI, figure 5D). The canal is anteriorly directed on the ventral side of the brain cavity and connects to the ventral side of the posterior myodome.

The vagus nerve (X, figure 5A,B,D) exits in the occipital region, right posteriorly to the acoustic capsule. From the left vagus nerve track, only a small part has been preserved, connected to the preserved fracture of the brain cavity. It is relatively broad and posterolaterally directed towards the lateral neurocranium wall. On the right side of the specimen also only a small part of the vagus nerve is preserved and it has a complex structure. The main body of the nerve canal reaches close to the lateral wall of the neurocranium, and from that point three canals branch off. The most anterior nerve track (X.lc, figure 5A,B,D) directs anteriorly and reaches very close to the lateral sensory line branch of the facial nerve (VII.lc, figure 5). The medial branch (X.ppl, figure 5A,B,D) is dorsally directed forming a groove in the wall of the neurocranium. This branch is following the direction of the posterior pitline and ends in the foramen of the endolymphatic duct, on the roof of the neurocranium. The third branch is posterolaterally oriented and exits the neurocranium from a foramen in the posterolateral wall in the occipital region. On the left side of the specimen, posterior to the vagus nerve two spino-occipital nerve canals exit the brain cavity. Both of them are directed towards the lateral wall of the neurocranium.

Anteriorly, in the ethmoid region, the hypophysial duct (hyp, figure 5A,B,D) opens dorsally in the preorbital area. The part of the endocranial cavity connected to the hypophysial duct is absent. The hypophysial duct is anteroventrally directed and opens in the mouth. The posterior wall of the hypophysial duct is connected to the hypophysial vein (hyp.v, figure 5A,B,D) which runs posteroventrally on the endocranial floor and connects to the pituitary vein. The pituitary vein (pit.v, figure 5) is laterally oriented, situated on the cranial floor, between the orbits in the anterior orbital region.

The otic capsule of *Romundina* sp. nov. is situated between the recess of the facial–acoustic nerve and the vagus nerve. The perichondral bone of the otic capsule is heavily fragmented and some of the features of the capsule have only been preserved on the left side of the specimen. From the impression of the preserved perichondral bone, the sacculus, on the ventral side of the capsule, appears to be bulky, occupying almost all the space between the cranial wall and the endocranial cavity (figure 5). The anterior ampullae, dorsal to the sacculus (amp.a, figure 5B), has a spherical shape and extends close to the orbit. From the anterior ampullae, the anterior semicircular canal extends dorsally close to the skull roof and turns posteriorly (csa, figure 5A), but only a small part of the canal has been preserved and it does not reach its full length. Posteriorly and slightly ventrally, the ampullae of the exterior canal (amp.e, figure 5B), seen from the anterior side, create a very small angle with the anterior ampullae (figure 5). On the posterior side of the capsule the posterior semicircular canal (csp, figure 5B,D) is dorsally directed from the sacculus. The canals of the endolymphatic system, are dorsally directed, connecting to the skull roof, in the occipital region (d.end, figure 5).

#### 3.2.4. Gnathal plates

CPW.9 displays on the ethmoid surface of the endocranium two gnathal plates considered, regarding their position, as anterior supragnathal plates (figure 2). They are quadrangular in shape and longer in their mesial part. Both plates are covered with internal odontodes of two kinds: overgrowing odontodes and teeth. Attachment of the gnathal plates to the perichondral ethmoidal bone, notably associated vascular spaces, was fully described by Smith *et al*. [37].

Gnathal plates are not preserved on MNHN.F.CPW30, but two horizontal bulges (figure 3J) are present on the ethmoid area where the plates should be located. Bulges of the perichondral bones underneath the gnathal plates are well observable on the virtual section through junction of premedian plate and anterior supragnathal plate ([37], fig. 1E).

##### 3.2.4.1. Overgrowing odontodes

**Figure 6.**
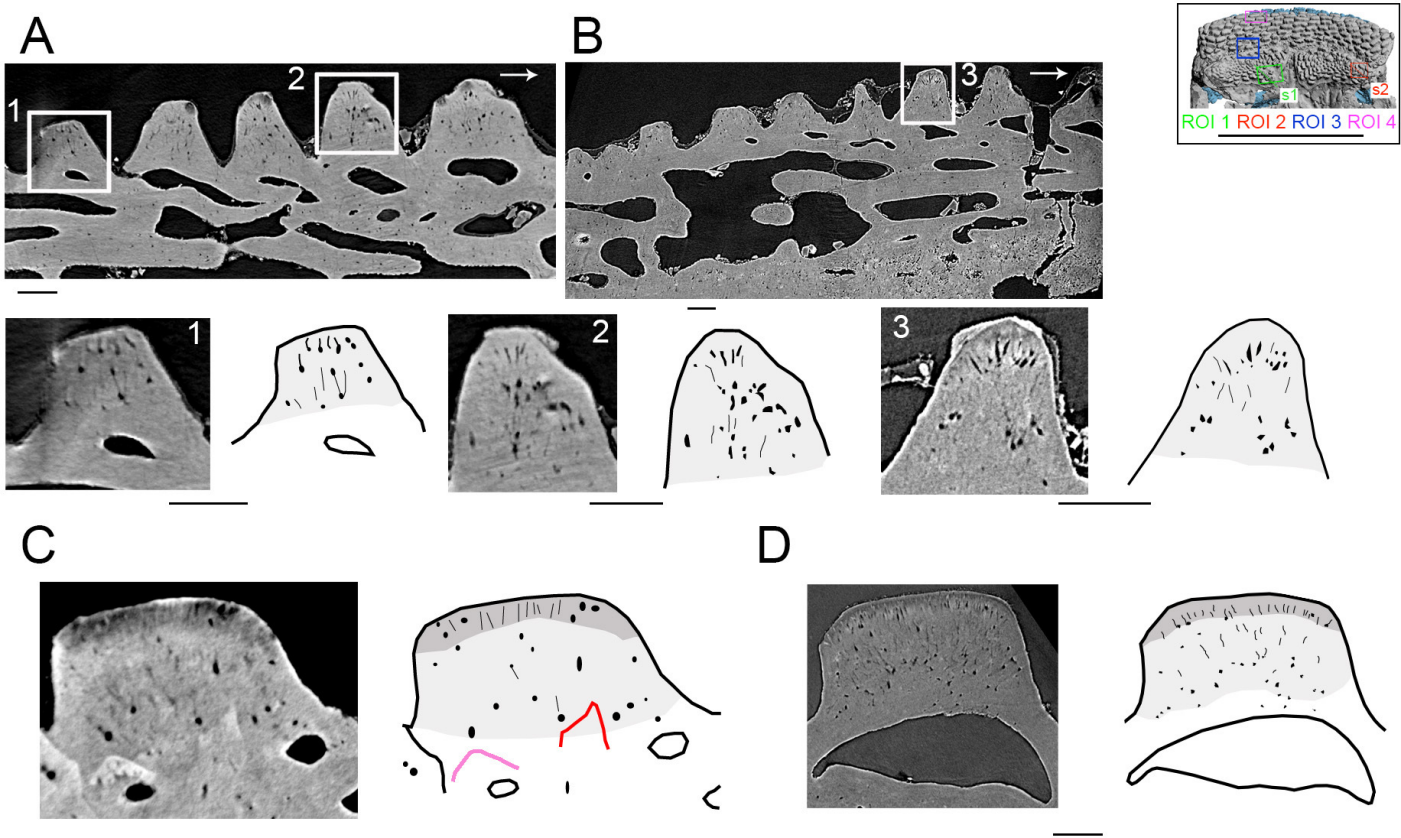
*R. gagnieri* sp. nov., histology, MNHN.F.CPW9. (A) SRXTM slice in the right gnathal plate (ROI 1, s1) with close-ups and interpretative drawings of two teeth. (B) SRXTM slice in the left gnathal plate (ROI 2, s2) with close-ups and interpretative drawing of one tooth. (C) SRXTM slice in one overgrowing odontode of the anterior area of the right gnathal plate (ROI 3) and interpretative drawing. (D) SRXTM slice in a dermal odontode of the ventral surface of the premedian plate (ROI 4) and interpretative drawing. Pale grey for the inner layer of semidentine, dark grey for the outer layer. Red odontode corresponds to a broken tooth. Pink odontode corresponds to an hypermineralized tooth. Arrows point posteriorly. Scale bars equal 100 µm. Miniature, on the top right, from figure 2 to locate ROIs (scale bar equals 1 cm).

**Figure 7.**
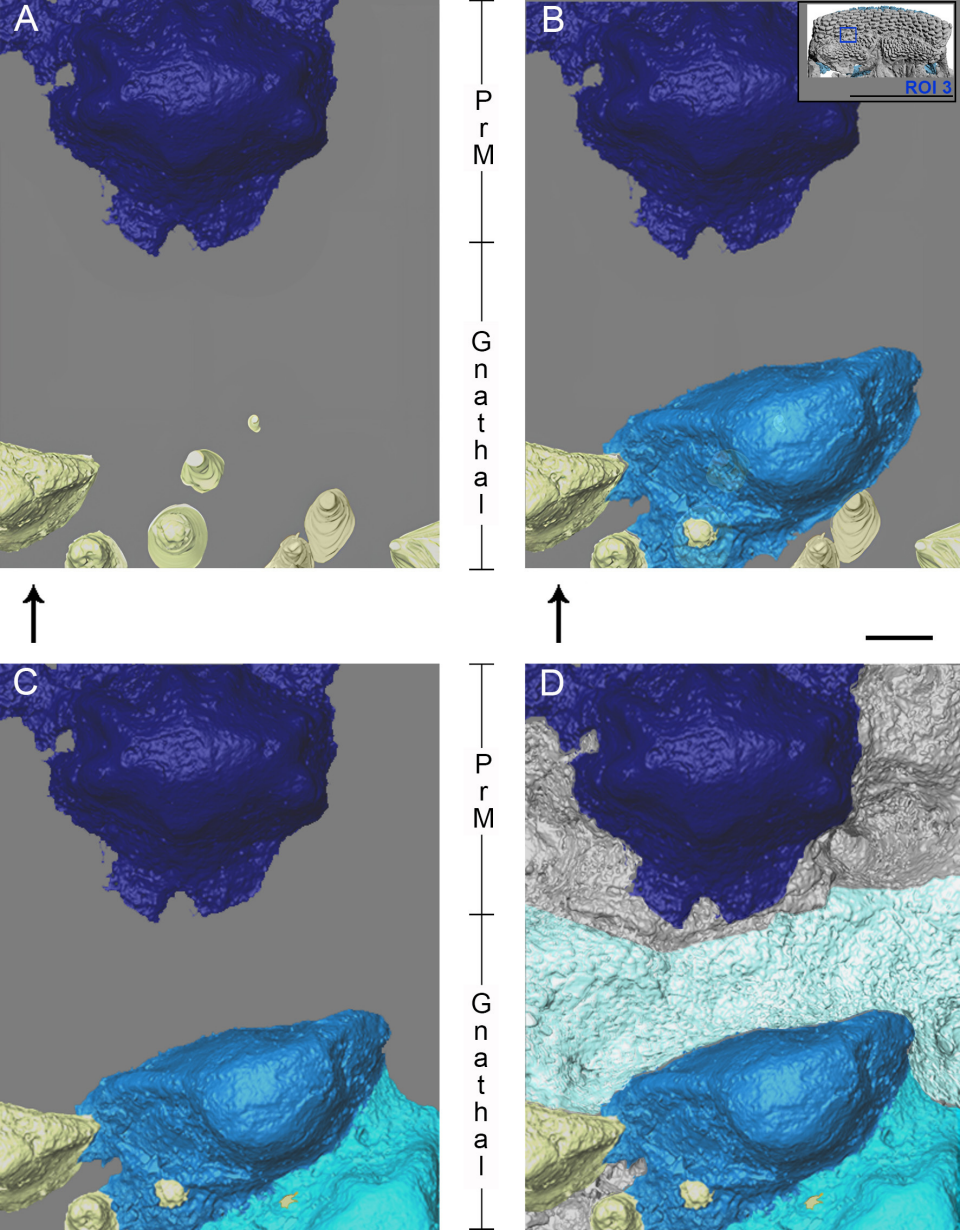
*R. gagnieri* sp. nov., overgrowing odontodes setting-up in the PrM/gnathal plate area (ROI 3), MNHN.F.CPW9, volume renderings of SRXTM data. (A) stage 1: gnathal plate with teeth and devoid of overgrowing odontodes. (B) stage 2: gnathal plate with the first generation of overgrowing odontodes (blue). (C) stage 3: gnathal plate with the first two generations of overgrowing odontodes (blue and cyan). (D) stage 4: gnathal plate with three generation of overgrowing odontodes/tissue (blue, cyan and light cyan). Arrows point anteriorly. Scale bars equal 0.1 mm. Miniature, on the top right, from figure 2 to locate ROI 3 (scale bar equals 1 cm).

**Figure 8.**
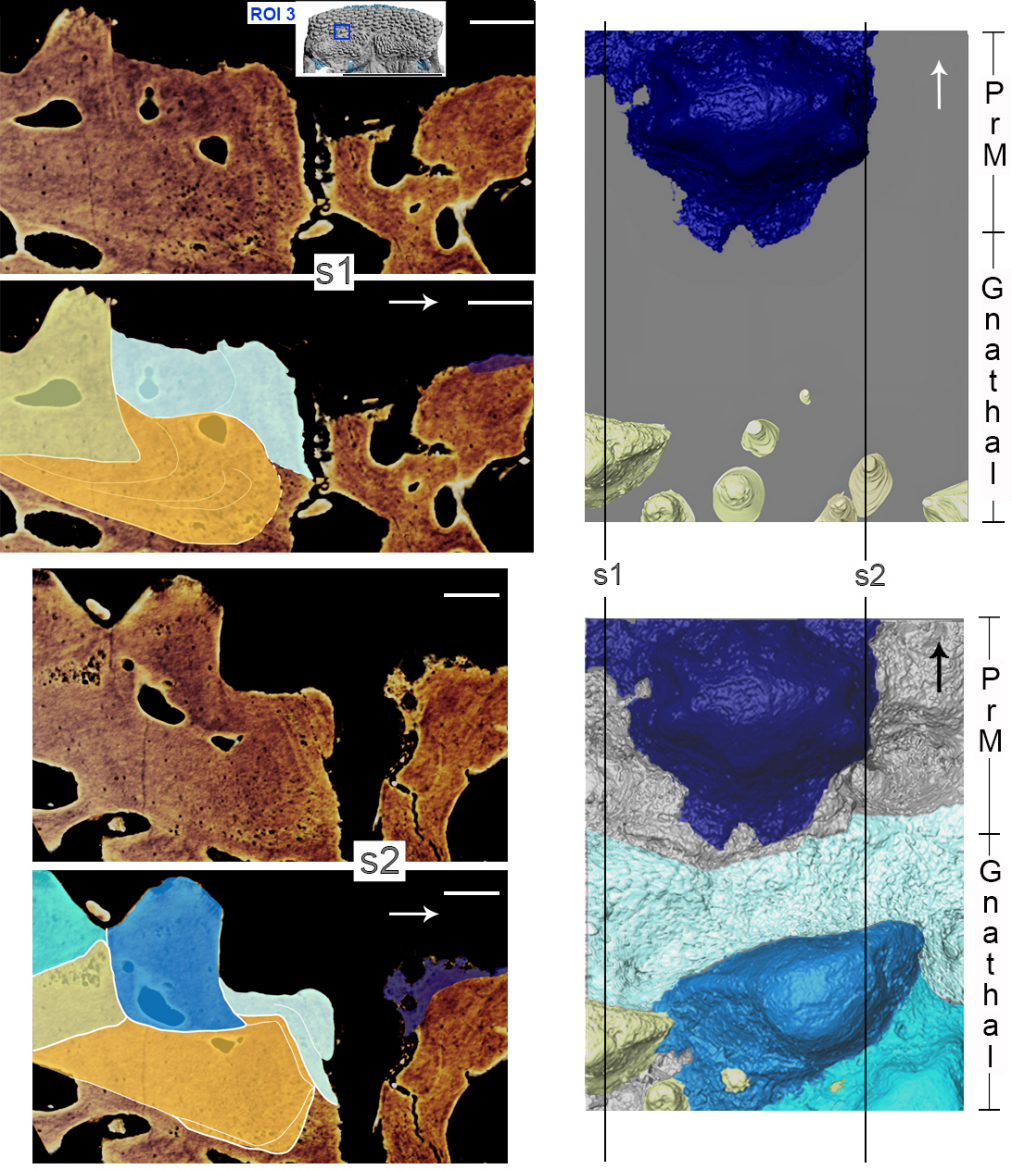
*R. gagnieri* sp. nov., anterior growth of the gnathal plate and covering of the gnathal plate, MNHN.F.CPW9. On the right: PrM/gnathal plate area without (top) and with (bottom) overgrowing odontodes (figure 7), volume renderings of SRXTM data. On the left: virtual thin sections with locations indicated by s1 (SRXTM slices from 60 to 70) and s2 (SRXTM slices from 778 to 788) in the volume rendering with overgrowing odontodes. Virtual thin sections without (top of each) and with (bottom of each) interpretation. Yellowish colours for teeth and sheets of dental tissues and blueish colours for overgrowing odontodes (dark blue for the external odontode of the PrM plate). Arrows point anteriorly. Scale bars equal 0.1 mm. Miniature, on the top, from figure 2 to locate ROI 3 (scale bar equals 1 cm).

Large odontodes, overgrowing smaller internal odontodes (teeth) in the anteromedial part of the gnathal plate in *R. gagnieri* sp. nov, have already been noted [12]. SRXTM and three-dimensional modelling of this area confirms this superposition exists (figure 2: ROI 3, figures 6C, 7 and 8) but also indicates, for the first time, that (i) these overgrowing odontodes can overgrow several teeth, even broken ones (figure 6C, tooth in red), (ii) they cover entirely or partially the teeth (figures 7 and 8), (iii) they can overlay between them (figures 7 and 8) and (iv) they grow on the anterior part of the gnathal plate in successive stages (figures 7 and 8).

##### 3.2.4.2. Growth of gnathal plate

**Figure 9.**
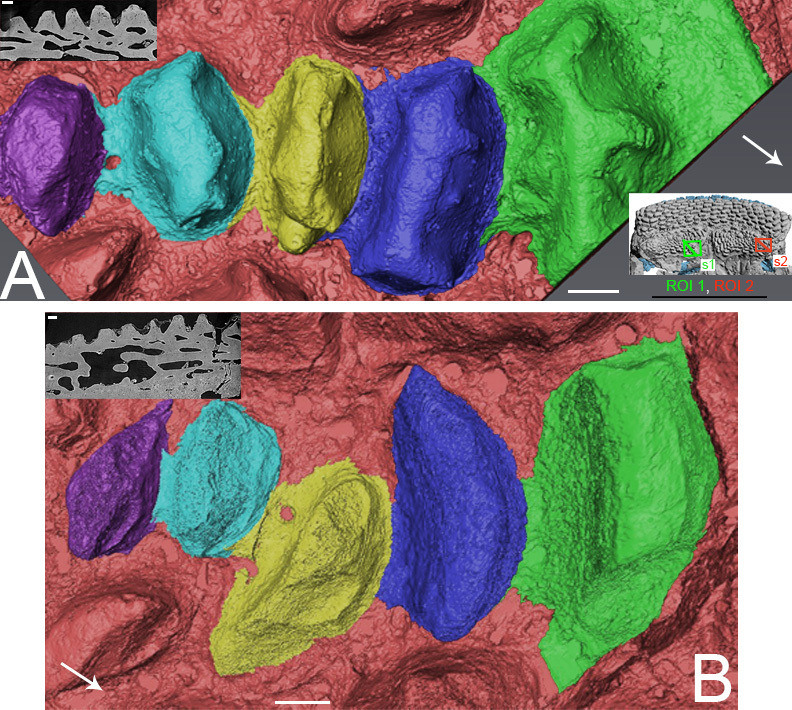
*R. gagnieri* sp. nov., centripetal growth of the gnathal plate, MNHN.F.CPW9. (A) volume rendering of SRXTM data of the teeth of figure 6A (miniature on the top left, s1 of figure 2), from the centre of the gnathal plate towards the medial teeth. (B) volume rendering of SRXTM data of the teeth of figure 6B (miniature on the top left, s2 of figure 2), from the centre of the gnathal plate towards the lateral teeth. Red represents parts of the gnathal plate that were not volume rendered in detail. Arrows point posteriorly. Scale bars equal 0.1 mm. Miniature, on the right, from figure 2 to locate ROIs 1 and 2 (scale bar equals 1 cm).

Teeth from the medial border towards the centre of the gnathal plate (figure 6A) display the same profiles as teeth going from the lateral border of the gnathal plate towards the centre (figure 6B), i.e. low and flat-top proximal teeth, and high and rounded distal teeth. The three-dimensional modelling of ROIs 1 and 2 (figure 9) reveal a centrifugal growth with newly added teeth accommodating the distal margin of the previous (older) ones.

On the gnathal plate, anterior teeth differ from medial and lateral teeth. They are lower and slenderer. Three-dimensional modelling in this area (figures 7 and 8) shows that there is an anterior tooth addition. Anterior and younger teeth/sheets of tissue accommodate the distal margin of the posterior and older ones (for instance, the posterior margin of the orange youngest tooth accommodating the anterior margin of the yellow older tooth, figure 8). The anterior tooth addition area is mainly covered by large overgrowing odontodes, making it difficult to observe, especially without SRXTM data.

### 3.2.5. Histology

Teeth, overgrowing odontodes and external odontodes are situated on a thick spongy bone layer (figure 6). External odontodes consist of two layers of dentine with numerous mainly unipolar odontocyte lacunae (=semidentine, figure 6C,D). The inner layer of semidentine displays dispersed lacunae and fills almost all of the odontodes. The outer layer of semidentine is thin and consists of parallel tubules with few lacunae, resembling the coronal layer of Gross [50]. This three-layer arrangement (spongy bone layer and two layers of semidentine) was already noticed for the stellate odontodes of *R. gagnieri* sp. nov. (*Romundina* sp. in Johanson & Smith [6]: fig. 16B,C) and is similar to the histology of the odontodes of *R. stellina* ([51], fig. 41; [52], fig. 3B) and of the arthrodires *Phlyctaenius acadicus* ([50], fig. 9B,E) and *Dicksonosteus arcticus* ([53], pl. 14, figs 4 and 5). Only one layer of semidentine is observable in the teeth of *R. gagnieri* sp. nov. (figure 6A,B).

Some of the overgrown teeth (figure 6C, pink tooth) exhibit an X-ray dense tissue that we interpret as enameloid. This hypermineralized layer is absent on teeth that are not overgrown and might characterize unworn teeth. Another important feature, observed underneath the overgrowing odontode, is the presence of a broken tooth (figure 6C, red tooth).

At the anterodorsal margin of the gnathal plate layers of lamellar bone are wrapping around the element demonstrating a growth around the element (figure 8, s1, s2). Sharpey’s fibres at the anterodorsal margin with elongated anteriorly directed cavities are visible (figure 8, s1, s2).

**Figure 10.**
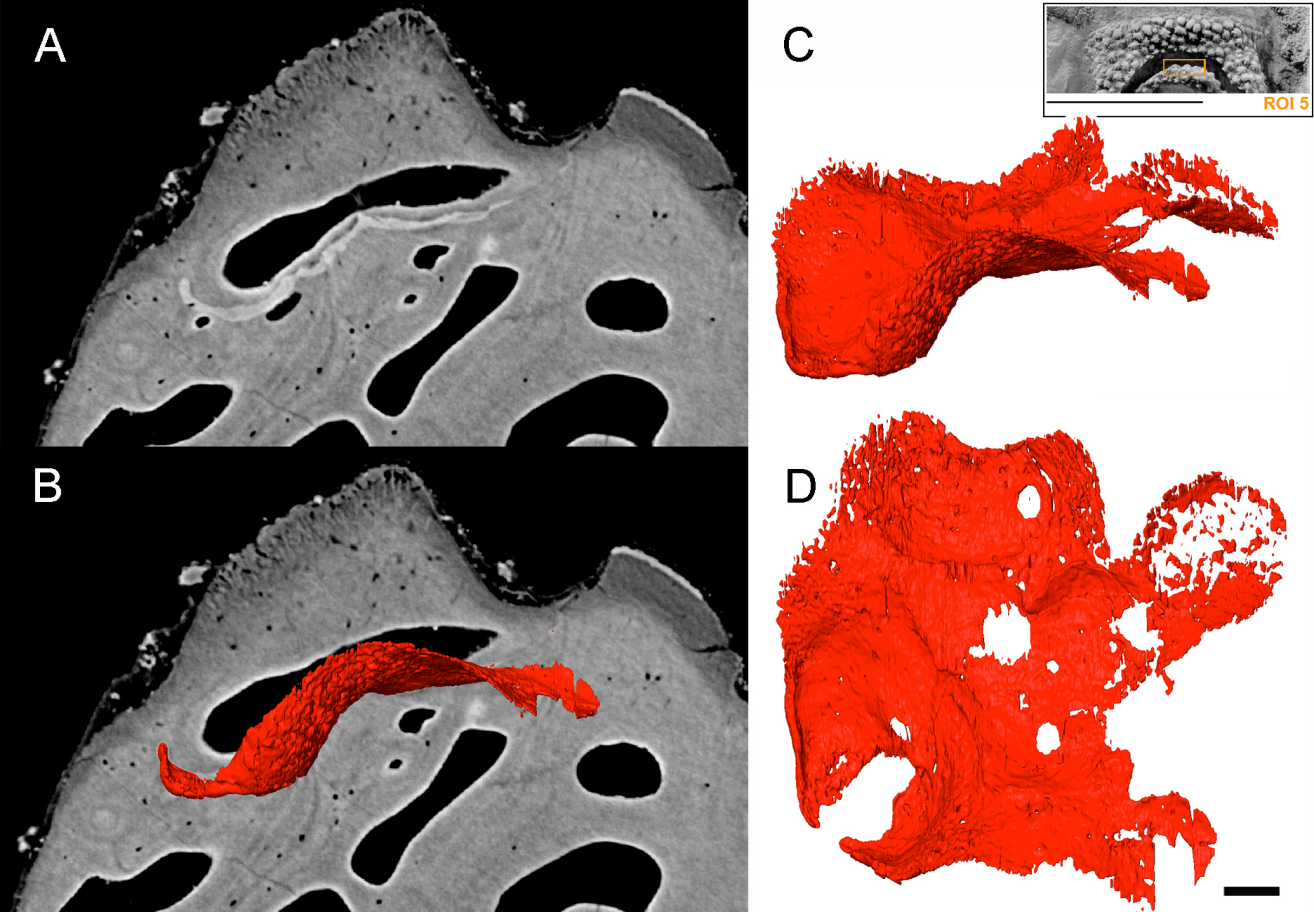
*R. gagnieri* sp. nov., resorption area at the ethmoid lateral line commissure (ROI 5), MNHN.F.CPW9. (A) ventro-dorsal SRXTM slice. (B) Ventro-dorsal SRXTM slice with the volume rendering of SRXTM data of the resorption cusps. (C, D). Volume rendering of the SRXTM data of the resorption cups in lateral (C) and dorsal (D) views. Scale bar equals 100 µm. Miniature, on the top right, from figure 2 to locate ROI 5 (scale bar equals 1 cm).

Growth and addition of odontodes is generally appositional to the dermal bones. At the ethmoid lateral line commissure on the dorsal side of the premedian plate (figure 2A, ROI 5), an X-ray dense layer is visible in the superficial layer underneath odontodes, truncating existing growth layers and odontodes, reflecting a resorption line. These form resorption cups and represent a rare example of remodelling of the superficial layer in the skeleton of *R. gagnieri* sp. nov. during ontogeny (figure 10), whereas remodelling the middle layer is a common mechanism in *Romundina* and other placoderms [54,55]. Resorption surfaces on gnathal elements have been described for arthrodires [6,56] and seem to be absent in acanthothoracids.

## 4. Discussion

### 4.1. *R. stellina* and *R. gagnieri* sp. nov

The adult skull of *R. gagnieri* sp. nov. is much longer (by extrapolation of what is preserved from MNHN.F.CPW9—a prenasal area of an adult specimen—compared to MNHN.F.CPW30—an incomplete skull of a juvenile specimen) than that of *R. stellina* (roughly 2 cm) but the juvenile skull of *R. gagnieri* sp. nov. is in the same size range as *R. stellina*. The ontogenetic stages of *R. stellina* are unknown; however, *R. stellina* differs from juvenile and adult specimens of *R. gagnieri* sp. nov. by (i) the ethmoid lateral line commissure that is not divided into two branches and that follows, on all its width, the anterior margin, as noted by Vaškaninová *et al*. [12, fig. S2, caption], (ii) the central sensory line groove and posterior pitline that point towards the centre of the skull roof and not anteriorly, (iii) the orbital area that is not constricted laterally; on the contrary, it increases progressively in width posteriorly, (iv) the absence of an anteroventral expansion of the premedian plate, (v) the orbital and premedian-ethmoid areas that are horizontal and not oriented dorsally as in *R. gagnieri* sp. nov., (vi) the skull that is less compressed laterally and the skull floor that is quite flat (strongly vaulted in *R. gagnieri* sp. nov.), and (vii) the branches of the cranial nerves that end up in the jaws (trigeminal, facial) with a direction more dorsally oriented. Two horizontal bulges supporting the anterior supragnathal plates are present on the ethmoid surface of the endocranium of *R. gagnieri* (figure 3J), but also of *R. stellina* [34, fig. 2 A2]. Thus, it is likely that *R. stellina* was also bearing anterior supragnathal plates, as suggested by Dupret *et al*. [34, p. 20].

Based on their morphology, it has been hypothesized that the earliest-branching placoderms were primarily benthic organisms [29]. Antiarchs and petalichthyids exhibit morphological characteristics that are congruent with that hypothesis [57,58]. The dorsal position of the eyes of *Brindabellaspis* [59] is an example of this kind of adaptation. However, in some species of ‘acanthothoracids’, the position of the eyes and their overall body morphology implies a more active swimming lifestyle [23]. The difference in the eye position between *R. gagnieri* sp. nov. and *R. stellina*, i.e. more lateral in *R. gagnieri* sp. nov., suggesting an ecological distinction, with *R. gagnieri* sp. nov. living in pelagic habitat and *R stellina* in benthic environments.

The forebrain of vertebrates participates in several adaptive responses such as locomotion, spatial cognition and navigation [60,61]. The forebrain of *Romundina* is very short anteriorly, similarly to that of ostracoderms [62]. Ostracoderms, based on their overall body morphology, have long been viewed as cumbersome bottom feeders, occupying mainly benthic niches. However, Ferron *et al*. [63,64], using computational fluid dynamics, demonstrated that osteostracans and galeaspids could have been capable of greater locomotive ability and ecological diversity than previously thought. This suggests that the small volume of the forebrain in *Romundina* sp. does not necessarily reflect a low locomotive ability, typical of bottom benthic organisms—and as agreed with the possible pelagic habitat of *R. gagnieri* sp. nov.

### 4.2. Dentition of *R. gagnieri* sp. nov

The dentition of *R. gagnieri* sp. nov. is only known from the supragnathal plates. Since they lie on the ethmoid part of the endocranium, they are considered as anterior supragnathal plates by homology with arthrodires where anterior and posterior supragnathal plates are present (the posterior ones lie on the palatoquadrate whereas the anterior ones lie on the ethmoid part of the endocranium). Unfortunately, the palatoquadrate and the suborbital plate of *R. gagnieri* sp. nov. are unknown. Thus, it is not yet possible, to know if posterior supragnathal plates were present, nor if a supplementary dentition (i.e. marginal) was contributing to the gnathal plate dentition. However, a marginal dentition is absent in *R. stellina* [16,34,65] and this is most likely the same condition in *R. gagnieri* sp. nov. The presence of a marginal dentition in ‘acanthothoracids’, and more precisely in *Radotina*, has recently been refuted [16].

As in other ‘acanthothoracids’, infragnathals have not been retrieved for *R. gagnieri* sp. nov. and the condition of the lower jaw in this group of placoderms remains unknown. Therefore, the two-upper-and-one lower pattern of jaw bones that supports the close relationship between arthrodires and maxillate placoderms plus crown gnathostomes [66–68], is not testable in the present case. What we know regarding the dentition of *R. gagnieri* sp. nov. is that it was composed of one pair of anterior supragnathals with teeth and that overgrowing odontodes colonized the oral cavity and were recruited as part of the dentition (see below).

### 4.3. Growth of the gnathal plate

On the anterior supragnathal plates of MNHN.F.CPW30, teeth are arranged in a radiating and concentric manner and centred on the anteromedial part of the plates [5,6,12,37] (but the first three papers did not recognize teeth and evoked modified versions of dermal tubercles or denticles). It was suggested that the anteromedial area of the gnathal plate could be the growth centre [12,37]. For Vaškaninová *et al*. [12, p. 3], the tooth addition is ‘radial from a labial founder region, and there is no labial tooth addition’, whereas it is radial and in all directions for other authors [5,6,37] (mistakenly indicated ‘centripetal growth’ instead of ‘centrifugal growth’). SRXTM data (figures 8 and 9) show that there is a radial/centrifugal tooth addition, including an anterior (labial) one. Central teeth are flat-topped and low. They correspond to abraded old teeth contrary to distal teeth that are higher, more rounded and correspond to newly added teeth (anterior teeth are lower and slenderer than medial and lateral teeth). The outer hypermineralized layer observed on one anterior tooth (figure 6C, pink tooth) is not observed on other teeth, i.e. central, medial and lateral teeth. This suggests that the newly formed anterior teeth are quickly covered by the overgrowing odontodes and are thus not abraded by food or occlusion during the lifetime of the animal (the putative broken tooth in the same area (figure 6C, red tooth) would therefore represent an early accident in the animal’s life). On the contrary, the oldest teeth in the centre of the gnathal plate and medial and lateral teeth (figure 6A2,B), that are not covered by overgrowing odontodes and that are thus active in the feeding process, could have lost the outer hypermineralized layer. Since the anterior supragnathal plates are fixed to the ethmoidal region of the endocranium in a later ontogenetic stage (MNHN.F.CPW9) and absent in the earlier one (MNHN.F.CPW30), it is likely that the anterior tooth addition is limited and stopped when it meets the anteroventral portion of the premedian plate. Evidence for a later fusion of the anterior supragnathal plate to the ethmoidal ossification are also layers of bone wrapping around the anterodorsal margin of the gnathal plate and Sharpey’s fibres showing a connection with muscles. Also, a layer of spheritic mineralization connects the plate to the ethmoidal ossification in the larger specimen demonstrating fusion later in ontogeny.

Regarding the overgrowing odontodes, their development on the gnathal plate occurs in successive steps without obvious organization (figures 7 and 8). We rationalize the pattern of morphogenesis as follows (figure 7): (i) the first step (figure 7A), the gnathal plate occurs with teeth free of covering, (ii) the first generation of overgrowing odontodes (figure 7B, blue) develops and is followed (because the anterior part of the second generation odontodes covers the posterior part of the first generation odontodes) by the addition of the second generation odontodes (figures 7C and 8, cyan odontode), (iii) finally, or maybe during the development of the second generation odontodes, another layer—or probably several thin layers—of bone (figure 7C, light cyan) develop on the first generation of overgrowing odontodes and on the uncovered teeth (figures 7D and 8). It is likely that this layer of bone originates from the front since it is only located anteriorly to odontodes that are the most extended dorso-ventrally on the gnathal plate (both teeth and overgrowing odontodes). Conversely, the absence of barrier (extended dorso-ventrally odontodes) permits its posterior progression (figure 8, right side of the blue odontode on the gnathal plate on the three-dimensional model). Vaškaninová *et al*. [12] inferred that overgrowing odontodes on MNHN.F.CPW9 were dermal in origin. This inference presumably rests with the similarity in morphology and volume of the overgrowing odontodes and the odontodes of the premedian plate (previously observed by Smith *et al*. [37]). This is insufficiently robust evidence on which to infer the origin of cells from which the odontode-forming tissues were derived. Also, our data document overgrowing odontodes that are added randomly in the anterior part of the gnathal plate and therefore not fitting with the suggested scenario of an overgrowth of dermal odontodes in the oral direction from the premedian plate.

The developmental relationship between teeth and external odontodes have been studied in stem osteichthyans [13,69] and basal sarcopterygians [70,71]. Although tooth development of osteichthyans differs greatly from placoderms in their tooth-shedding ability (e.g. [72]), some comparisons are possible with stem osteichthyans which also lack tooth rows and possess teeth and external odontodes of similar sizes [8,13,69]. Recently, the dental ontogeny of the stem osteichthyan *Lophosteus superbus* and the developmental relationships between its teeth and dermal odontodes have been studied in detail [13]. It was proposed that teeth and external odontodes are modifications of a single odontode system and that the initiation site for the differentiation of odontodes between teeth and external odontodes is located on a marginal jawbone. The initiation site is inferred to be centred on two longitudinal ridges of odontodes from which external odontodes and teeth were added sequentially in opposing directions, attaining a stellate and conical morphology, respectively. In *Lophosteus*, ‘regulatory cross-contamination’ has been inferred at the invasion zone between external odontodes and teeth [13]. The latter implies that, in this area, the teeth have the character of external dermal odontodes while the external odontodes have a tooth-like character. Unfortunately, regulatory cross-contamination is not a phenomenon that can be observed in fossils.

### 4.4. ‘Acanthothoracid’ dentitions and ancestral condition for vertebrate tooth growth

Placoderms constitute the sister clade [21,22] or grade [18–20] to the monophyletic group comprised of osteichthyans and chondrichthyans. Among placoderms, the dentition has only been known in detail from arthrodires and ptyctodonts [1,2,6,49,51,53]. Thus, it was argued that the arthrodires, with their nonmarginal dentition with a defined and restricted number of radially arranged tooth files with an anterior (labial), posterior (lingual) or longitudinal addition, might represent a primitive condition for jawed vertebrates [2].

This view has been challenged by the discovery of a statodont dentition carried by marginal dermal bones in ‘acanthothoracids’ [12]. Here, teeth exhibit posterior (proximal) sequential addition and they are not shed. Given the phylogenetic position of ‘acanthothoracids’ inferred by some [12,19,67], and the distribution of this dentition character in the gnathostome tree, this developmental mode has been inferred as plesiomorphic for jawed vertebrates [12,73]. Furthermore, and independently, arthrodiran upper gnathal plates have been interpreted as homologous to the palatal laminae of marginal jaw bones (with the loss of facial laminae in arthrodires) of a gnathostome ancestor which possessed facial and palatal laminae [67,68], emphasizing the derived character of arthrodiran condition [22,68].

However, the existence of a marginal dentition in ‘acanthothoracids’ (i.e. in *Radotina*) has recently been refuted and with it, the proposal that it reflects the primitive condition for the dentitions of jawed vertebrates [16]. Considering the jaw hinge, it appears that ‘acanthothoracid’ dentitions were fundamentally similar in location to the dentitions of arthrodires, rather than resembling bony fishes [16]. The dentition in *R. gagnieri* sp. nov. (known as CPW.9 in [12]) exhibits tooth addition not only posteriorly (lingually), but also anteriorly (labially), on bones that are not marginal but rest on the ethmoid surface of the endocranium, making them gnathal plates, a condition shared with arthrodires [5]. Moreover, the pattern of sequential addition of the teeth in *R. gagnieri* sp. nov. (partially shown by Vaškaninová *et al*. [12, fig. S3] and clarified here in figures 8 and 9) is very similar to that seen in arthrodires which possess fewer rows on the upper gnathal plates. Thus, an increasing number of features appear to support a phylogenetic relationship between arthrodires and ‘acanthothoracids’. This may indicate not only that jaw morphology was phylogenetically conserved across most placoderms [16], but so was the inner position of the gnathal elements. This returns us full circle to the starting historical hypothesis, that the dental condition in arthrodires and now ‘acanthothoracids’, reflects the gnathostome ancestral condition [51].

Interpreting these data is challenged by the uncertainty over whether placoderms comprise a clade or an evolutionary grade, which we cannot resolve here. If we assume that placoderms are monophyletic, comprising the sister clade of osteichthyans and chondrichthyans [5,21,22] our observations on the growth of the gnathal plate in *R. gagnieri* sp. nov. argue for an early loss of the facial lamina of the marginal jaw bone in the history of placoderms. Previously considered as a synapomorphy of arthrodires [22], it is now considered as a synapomorphy shared by all core placoderms (excluding maxillate placoderms, *sensu* [22]).

## 5. Conclusion

The description of a new species of the ‘acanthothoracid’ genus *Romundina* adds to the gnathostome diversity of the Early Devonian of Arctic Canada. The two species were contemporaneous and probably occupied different ecological niches (pelagic and benthic, respectively for *R. gagnieri* sp. nov and *R. stellina*). The description of *R. gagnieri* sp. nov. allowed us to better understand the growth pattern of upper jaws in ‘acanthothoracids’ thanks to SRXTM. This study showed that teeth that are developed first during the ontogeny of *R. gagnieri* sp. nov. had a hypermineralised layer, they can be broken and overgrown by odontodes later during ontogeny. Overgrowing odontodes are present on the anterior part of the gnathal plates and are added without obvious organization and in several stages. Overgrowing odontodes colonized the oral cavity and participate in the feeding process. A sheet of lamellar bone is extending on the anterodorsal side of the gnathal plate. Together with Sharpey’s fibres, they demonstrate that the plate was not fixed to the ethmoidal ossification early on during ontogeny and is only later connected through mineralizations.

The morphology and function of ‘acanthothoracid’ jaws [16], as well as the presence of a pair of anterior supragnathal plates on the ethmoid part of the endocranium of the new ‘acanthothoracid’ taxon *R. gagnieri* sp. nov., and together with the growth pattern of these plates in this taxon (centrifugal with an anterior tooth addition, e.g. [49]), all resemble generalized placoderm conditions seen notably in arthrodires. These similarities argue for a phylogenetic conservation of the jaw structure during the placoderm evolutionary history. In the context of osteichthyan evolution, and considering ‘placoderms’ as paraphyletic, they indicate that the gnathostome ancestral condition may be represented by the common dental condition seen in arthrodires and now ‘acanthothoracids’. More robust phylogenetic hypotheses [16,20,21,67,74], together with discoveries of new placoderm taxa displaying elements of the jaws, will clarify the situation in the near future.

## Data Availability

Raw slice data are available in the Dryad Data Repository [40].
